# Synthesis and Biological Evaluation of 6-*O*-Sucrose Monoester Glycolipids as Possible New Antifungal Agents

**DOI:** 10.3390/ph16020136

**Published:** 2023-01-17

**Authors:** Michele Verboni, Maurizio Sisti, Raffaella Campana, Serena Benedetti, Francesco Palma, Lucia Potenza, Simone Lucarini, Andrea Duranti

**Affiliations:** Department of Biomolecular Sciences, University of Urbino Carlo Bo, 61029 Urbino, PU, Italy

**Keywords:** glycolipids, sucrose monoesters, sugar-based surfactants, minimum inhibitory concentration (MIC), antifungal activity, anti-inflammatory activity

## Abstract

A small library of 6-*O*-sucrose monoester surfactants has been synthesized and tested against various microorganisms. The synthetic procedure involved a modified Mitsunobu reaction, which showed improved results compared to those present in the literature (higher yields and larger scope). The antifungal activities of most of these glycolipids were satisfactory. In particular, sucrose palmitoleate (URB1537) showed good activity against Candida albicans ATCC 10231, *Fusarium* spp., and *Aspergillus fumigatus* IDRAH01 (MIC value: 16, 32, 64 µg/mL, respectively), and was further characterized through radical scavenging, anti-inflammatory, and biocompatibility tests. URB1537 has been shown to control the inflammatory response and to have a safe profile.

## 1. Introduction

Fungal invasive infections represent a serious problem in human health and are associated with at least 1.5–2 million deaths worldwide [1,2,3,4]. In this scenario, opportunistic mycotic pathogens such as *Candida* spp., *A. fumigatus* and *C. neoformans* are the most important causes of invasive fungal infections, accounting for more than 90% of fungal deaths [4]. These infections are mainly common in immunocompromised patients subjected to anticancer therapy, long-term corticosteroid treatments or organ transplant as well as immunosuppressive infections. Moreover, fungi are responsible for infections on skin and mucosal surfaces with a greater incidence compared to invasive fungal ones. In addition, the development of antifungal drugs faces off with the fact that fungi are eukaryotes as their hosts and thus the potential targets may also be found in human cells with a substantial toxicity risk. Nowadays, the antifungals on the market are represented by various classes of drugs called polyenes (i.e., amphotericin B), azoles (i.e., fluconazole), allyl-/benzylamines (i.e., terbinafine), benzofurancyclohexenes (i.e., griseofulvin), echinocandins (i.e., caspofungin), antimetabolites (flucytosine), nikkomycines (nikkomycine Z), thiocarbamates (tolnaftate), and morpholines (amorolfine) [5,6]. Unfortunately, most of the antifungals and antibiotics actually commercially available, in particular those systemically and *per os* administered, show some limits, such as toxicity, the spectrum of activity, safety, pharmacokinetic properties, and drug-resistant strains [7]. Consequently, there is an urgent need to develop new antifungals as well as new antibiotics to fill this therapeutic gap.

Sucrose fatty acid esters (SFAE) are the most common glycolipids derived from polyalcohol sucrose (polar head) and naturally occurring fatty acid (hydrophobic tail). They are non-ionic surfactants and considered “green” because they are non-toxic and biodegradable. Moreover, they are non-irritant, odorless, and possess a safer biocompatibility profile in comparison with other amphiphiles such as polysorbates and sulfates [8]. Most of the time, SFAEs are synthesized from inexpensive and renewable agricultural products [8,9], and some of them are commercially available [8]. Moreover, they possess excellent emulsifying characteristics [10,11,12], antibacterial [8,10,13], antifungal [8] and insecticidal [8,14] activities, and permeability-enhancing properties [10]. For all these reasons, sucrose esters are very important commodity compounds in the agriculture, food, nutraceutical, cosmetic, dental, and pharmaceutical industries. In the 2010s, the world production of SFAE was above 6000 tons per year [8] and their market is projected to grow from USD 76 million in 2019 to USD 106 million by 2025 [15].

As part of our ongoing investigations on the synthesis, biological activities, and applications of sugar derivatives [16,17,18,19,20,21,22,23,24], a series of 6-*O*-sucrose monoesters were designed and synthesized by using an opportunely modified Mitsunobu-type methodology [25] starting from free sucrose and lipophilic acids. This coupling, compared to the others reported in the literature [8,10], such as esterification (acyl chlorides, anhydrides) and transesterification with activated esters (vinyl or enol esters) [26,27,28,29], and/or enzymatic preparation methods [30,31,32], showed higher conversions/yields, high regioselectivity and larger scope including saturated, mono- and polyunsaturated fatty acids or aromatic and alkyl aromatic acids.

The small library of sucrose esters was explored for antibacterial and antifungal activities and the profile of the most interesting glycolipid (URB1537) was deepened through the study of its antioxidant, anti-inflammatory, and biocompatibility properties.

## 2. Results and Discussion

### 2.1. Chemistry

A series of glycolipids characterized by the 6-*O*-sucrose hydrophilic head and a lipophilic tail of various sizes and nature has been designed and synthesized with the aim of exploring the influence of the hydrophobic portion on their biological properties. For this purpose, both linear saturated (caprylic (C8)), monounsaturated (undecylenic (C11:1), undecylinic (C11:1), palmitoleic (C16:1), oleic (C18:1), and nervonic (C24:1)) or polyunsaturated (linoleic (C18:2) and linolenic (C18:3)), and aromatic (benzoic and *p*-phenylbenzoic) or alkyl aromatic (phenyl-, *p*-biphenyl- and *p*-triphenylacetic) fatty acid derivatives substituents have been used. The most important physicochemical constants in the landscape of surfactants are hydrophilic–lipophilic balance (HLB), octanol–water portion coefficient (logP) and the polar surface area (TPSA). However, TPSA could not constitute a discriminant indicator when the polar head is maintained constant in all the designed surfactants, as in our case (calculated TPSA = 196 Å^2^ for sucrose esters). The glycolipids were indeed designed with the aim of keeping the HLB and logP values in a non-strict range to try to understand if a relationship between the biological activity and physicochemical properties is present.

With the only exception of sucrose nervonate (HLB = 8.6, Table 1, Entry 8), all the derivatives have HLB values greater than 9.5 (9.7–13.4) classifying the molecules as hydrophilic surfactants and are consequently able to act as oil-in-water emulsifiers. On the other hand, the designed sucrose-based glycolipids are molecules showing a widespread calculated logP (clogP) ranging from a negative value −2.7 (sucrose benzoate, Entry 9) to a positive 2.9 (sucrose oleate, Entry 5). As above, the only exception of the series is the sucrose nervonate that showed a clogP out of the Lipinski’s rules of five (5.9, Entry 8) due to its very lipophilic long tail.

**Scheme 1:**
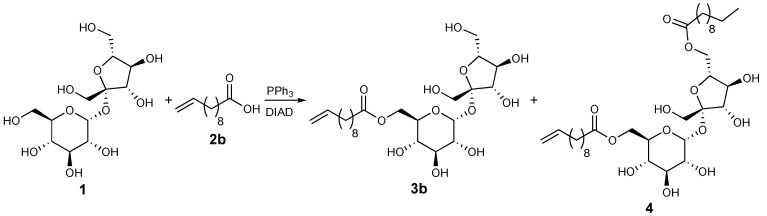
Model reaction for methodology studies.

The designed 6-*O*-sucrose-based monoesters could be synthesized by several reported procedures starting from unprotected sucrose [8,10]. We tried several of them by using classical chemical esterification and transesterification procedures and also using appropriate enzymatic methodologies but without satisfactory results in terms of regioselectivity and conversion/yield (data not shown). On the contrary, the Mitsunobu methodology reported by Molinier et al. [25] immediately proved to be suitable for our purposes. Indeed, not protected sucrose (**1**) and undecylenic acid (C11:1) (**2b**) under the reported Mitsunobu conditions gave the corresponding 6-*O*-sucrose ester **3b** in 34% yield (Table 1, Scheme 1 and Entry 1) and other known side products including the diester **4** [25]. With the aim of ameliorating the yield of **3b**, the reaction reported in Scheme 1 was studied as a model reaction. All the conditions were investigated in detail and the results are reported in Table 2.

As reported in Table 2 (Entry 1), the diester **4** represents the main product of the first attempt (38% yield vs. 34% of the desired product). A reduction of the acid could be decisive, with 1 equivalent of **2b**, almost no diester was obtained; however, the yield of 6-*O*-sucrose monoester was reduced to 19% (Entry 2). A 1.5 equivalent of the model acid gave the best result in term of yield of the desired product **3b** (39%, Entry 3) and lower production of 4 (15%). With the aim to reduce the 6-*O*-undecylenyl-3′,6′-anhydrosucrose side product [25], the equivalents of the Mitsunobu reagents DIAD and PPh_3_ were also decreased (Entries 4 and 5). However, no good results were obtained. The yield of **3b** did not also increase with a higher amount of DIAD and PPh_3_ (Entry 6). Increasing the temperature (Entry 7) or the reaction time to 48 h (data not shown) did not ameliorate the production of **3b** but enhanced the formation of the diester **4**. Moreover, the reduction of the reaction time worsened the yield of the desired monoester (Entries 8 and 9). Finally, other polar aprotic solvents, such as THF and dioxane, did not solubilize well sucrose and the reaction gave only a trace amount of the product (Entries 10 and 11). Other azodicarboxylates were also used under optimal reaction conditions as comparison with DIAD. By the use of DEAD, the reaction gave the desired product **3b** with comparable yield, albeit slightly lower, but with a higher production of diester **4** (Entry 12). Instead, the use of DCAD led to a clear lowering of the yield of 6-*O*-sucrose undecylenate (Entry 13).

**Scheme 2:**
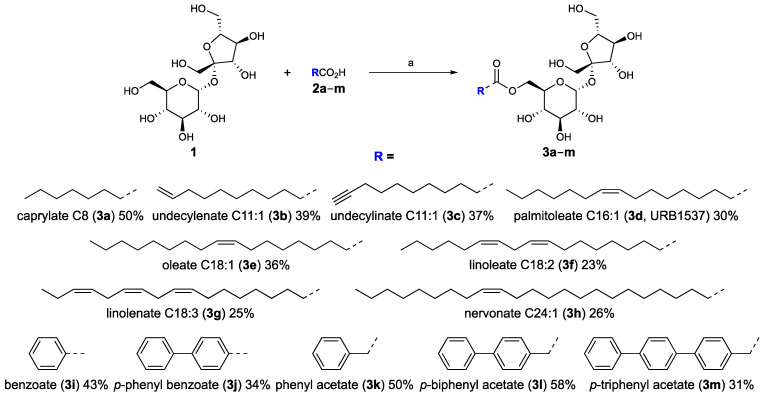
Sucrose ester synthesis. Substrate scopes, reagents, conditions, and yields: (a) PPh_3_, DIAD, dry DMF, rt, 24–30 h.

The improved Mitsunobu-type procedure (Entry 3) was then used to synthesize all the designed 6-*O*-sucrose monoester surfactants starting from not protected sucrose and the corresponding commercially available acids (Scheme 2).

The ameliorated methodology worked well for all the acid substrates giving the corresponding products in low to moderate yields (Scheme 2). Although the yields for some substrates are not exciting, it should be noted that the procedure uses commercially available, inactivated, or unprotected reagents, avoiding the activation or protection and deprotection steps. Moreover, the reaction is regioselective for the six-position of sucrose and, conveniently, the desired products are easily purified from the side ones by a short flash column chromatography. Therefore, this improved Mitsunobu-type esterification has shown to be versatile and has widespread substrate scope, and could be used for the production of any kind of 6-*O*-sucrose monoesters in low to medium scale.

### 2.2. Antibacterial Activity

The antimicrobial activity of the different sucrose esters is summarized in Table 3**.** As shown, the activity of the tested compounds was prevalently observed against Gram-positive bacteria (*Enterococcus faecalis* ATCC 29212, *Staphylococcus aureus* ATCC 43387, *S. aureus* ATCC 43300, *Listeria monocytogenes* ATCC 7644) but not against Gram-negative ones (*E. coli* O157:H7 ATCC 35150, *Klebsiella pneumoniae* ATCC 13883, *Pseudomonas aeruginosa* ATCC 9027, *Salmonella enteritidis* ATCC 13076). In detail, sucrose palmitoleate (**3d**) and sucrose oleate (**3e**) showed minimum inhibitory concentration (MIC) values of 1024 µg/mL against *E. faecalis* ATCC 29212, *S. aureus* ATCC 43387, *S. aureus* ATCC 43300 (MRSA) and *L. monocytogenes* ATCC 7644, while sucrose linoleate (**3f**) and sucrose linolenate (**3g**) reached MIC of 256 µg/mL and 512 µg/mL against *E. faecalis* ATCC 29212 and 1024 µg/mL against the other Gram-positive microorganisms. Sucrose nervonate (**3h**), sucrose benzoate (**3i**), and sucrose phenyl acetate (**3k**) were active only against *E. faecalis* ATCC 29212 (MIC 1024 µg/mL), while sucrose p-phenyl benzoate (**3j**) and sucrose undecylenate (**3b**) were active also against *L. monocytogenes* ATCC 7644 (in both cases MIC 1024 µg/mL). Sucrose caprylate (**3a**), sucrose undecylenate (**3c**), p-biphenyl acetate (**3l**), and p-triphenyl acetate (**3m**) were always inactive.

The mechanism of antibacterial action of sugar fatty acid esters is mainly based on the increase in permeability of the cell membrane and the consequent leakage of some cellular constituents, in particular proteins and sugars [35]. Polar compounds interact better with the bacterial cell wall, especially in Gram-positive bacteria, causing their damage. Effectively, our data showed that Gram-negative bacteria were more resistant to the examined sucrose esters compared to Gram-positive bacteria, probably due to their outer membrane, which limits the diffusion of sucrose fatty acid esters through the lipopolysaccharide covering. On the other hand, the literature data reports that the antibacterial activity of sugar fatty acid esters decreases rapidly as the chain length of fatty acid increases [36]. However, such a situation is not appreciable in this study because of the low activities of our tested compounds. Moreover, for the same reason, it is not possible to rationalize a correlation between the activity of the compounds and their physicochemical characteristics and lipophilic tails.

### 2.3. Antifungal Activity

The antifungal activity of the different sucrose esters is illustrated in Table 4. As a general trend, the tested compounds showed good antifungal activity and, interestingly, some of them (**3b**, **3d**–**f**) resulted active against all the selected fungi with MIC ranging from 16 to 1024 µg/mL. Among the molecules analyzed, the lowest MIC value of 16 µg/mL was observed for sucrose palmitoleate (C16:1) (**3d**), **3e** against *Candida albicans* ATCC 10231 and *Aspergillus fumigatus* IDRAH01, respectively, followed by **3f** (MIC 32 µg/mL against *A. fumigatus* IDRAH01). Further, **3b** resulted active against *Aspergillus niger* ATCC 9642 and *Fusarium* spp. (MIC 512 µg/mL) and **3g** showed MIC values of 128 µg/mL and 512 µg/mL for *Fusarium* spp. and *C. albicans* ATCC 10231, respectively. Similarly, **3i** and **3k** were active against *Fusarium* spp. and *C. albicans* ATCC 10231 with a MIC ranging from 256 to 1024 µg/mL. These two microorganisms showed to be sensitive also to the remaining sucrose esters, specifically **3b**, **3j**, **3l** (MIC 1024 µg/mL), confirming **3b** antimicrobial activity against *C. albicans*, reported in some previous research [37] As observed for bacteria, **3m** was inactive against all the examined fungi.

The best series of these sucrose esters against the tested fungi were the mono and polyunsaturated fatty acid-containing tail compounds. In detail, **3d** showed a relevant activity against all four fungi strains (MIC from 16 to 512 µg/mL). By decreasing the chain length, **3b** is less active showing higher MIC in the range of 512–1024 µg/mL. The molecule obtained by substitution of the terminal double bond with a triple bond, that is **3c**, was detrimental to the activity (MIC 1024 or >1024 µg/mL). From a general point of view, an increase in chain length seems to produce sucrose ester surfactants less effective against the tested fungi strains. Indeed, **3e–h** showed higher MIC values than **3d** with only one exception. Indeed, **3e** and **3f** seem selective and very active against *A. fumigatus* (MIC 16 and 32 µg/mL, respectively). The only saturated fatty acid containing tail compound, that is **3a** is less effective than **3d** (MIC 1024 µg/mL or greater). Alkyl aromatic and aromatic sucrose esters (**3i–m**) are also less effective against the tested fungi compared to **3d**. They showed MIC of 1024 or >1024 µg/mL with the only exception of **3i** and **3k** which showed interesting activities against Fusarium spp. (MIC 512 and 256 µg/mL, respectively), and *C. albicans* (MIC 256 and 1024 µg/mL, respectively), strains.

### 2.4. Radical Scavenging Activity

**Figure 1:**
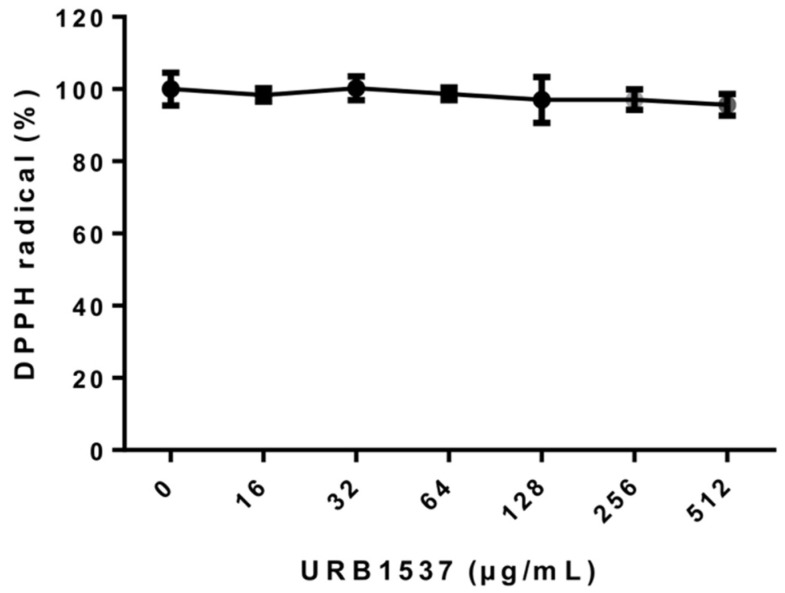
Evaluation of URB1537 scavenger activity against DPPH radicals. Data are expressed as the mean ± SD (*n* = 3).

The antioxidant activity of sucrose palmitoleate (**3d**, URB1537) was investigated by the 2,2-diphenyl-1-picrylhydrazyl hydrate (DPPH) radical scavenging assay. As indicated in Figure 1, URB1537 did not show scavenger effects against DPPH radicals within the range of concentrations tested (EC_50_ >> 512 µg/mL). This evidence agrees with previous findings showing that sugar esters possess lower radical scavenging properties than the reference antioxidant molecules [24,38,39], such as quercetin (calculated EC_50_ equal to 1.74 µg/mL). In particular, sugar esters show scavenger abilities at concentrations ranging from 1 to 10 mg/mL, higher than those herein tested for URB1537 (up to 512 µg/mL).

### 2.5. Biocompatibility Assay

**Figure 2:**
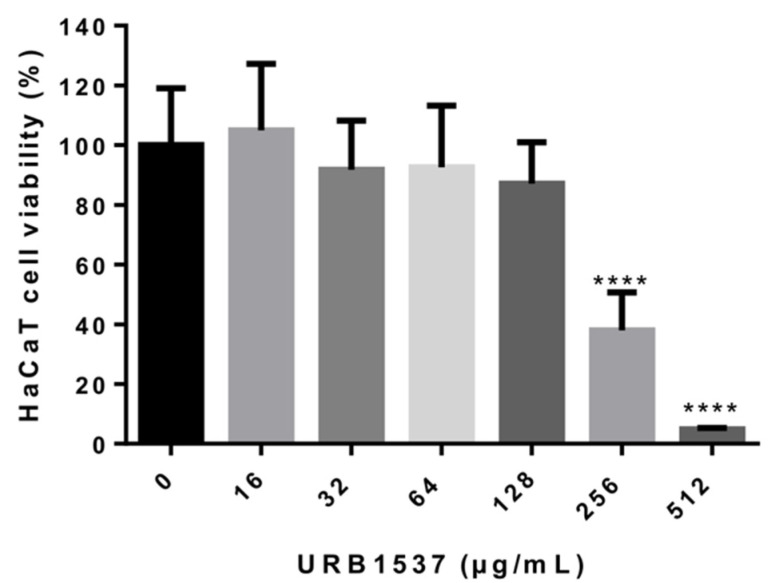
Cell viability evaluation after URB1537 administration to HaCaT cells for 24 h. Data are expressed as the mean ± SD (*n* = 3). **** *p* < 0.0001 vs. untreated control cells (Tukey’s post hoc test).

The evaluation of URB1537 cytotoxicity by 3-(4,5-dimethylthiazol-2-yl)-2,5-diphenyltetrazolium bromide (MTT) assay revealed a significant reduction of cultured human keratinocyte (HaCaT) cell viability with 256 and 512 µg/mL concentrations (Figure 2). The calculated IC_50_ value was equal to 230 µg/mL. Based on cytotoxicity criteria, URB1537 could be classified as weakly cytotoxic [40]. This finding confirms that both carbon chain length and sucrose ester concentration may influence cell viability, as previously evidenced for lactose-based fatty acid monoesters [19]. However, the cytotoxicity of URB1537 in HaCaT is still higher than the MIC showed against most of the selected fungi strains and the compound is indeed safe at least up to 128 µg/mL.

### 2.6. Anti-Inflammatory Activity

**Figure 3:**
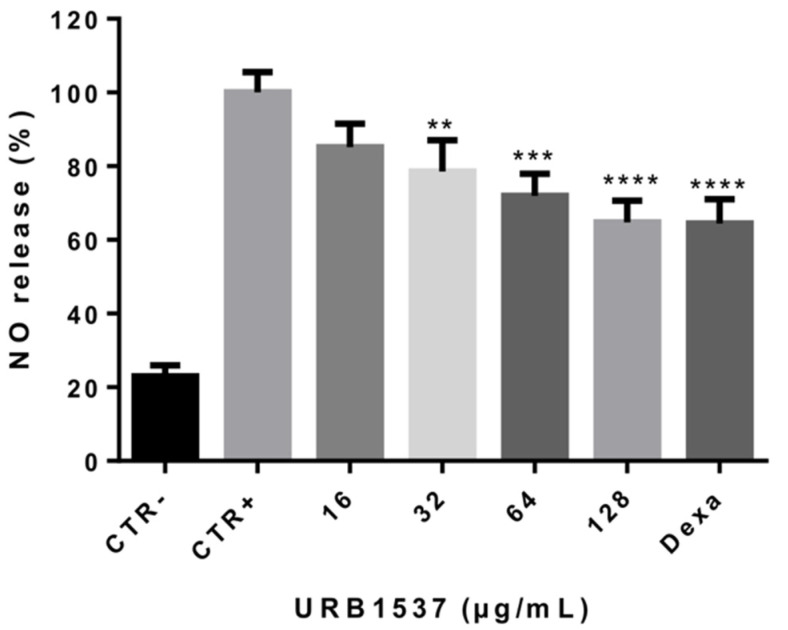
Extracellular NO release after RAW 264.7 stimulation by LPS for 24 h in the presence of URB1537 (16–128 µg/mL). CTR-: negative control (untreated cells); CTR+: positive control (LPS); Dexa: dexamethasone 2 µg/mL. Data are expressed as the mean ± SD (*n* = 3). ** *p* < 0.01, *** *p* < 0.001, **** *p* < 0.0001 vs. CTR+ (Tukey’s post hoc test).

As reported in Figure 3, the stimulation of RAW 264.7 cells (murine macrophages) by lipopolysaccharide (LPS) (CTR+) led to a strong extracellular release of the inflammatory response mediator nitric oxide (NO) as compared to untreated control cells (CTR-). When LPS-exposed cells were co-incubated with URB1537, a dose-dependent decrement of NO production was observed. The anti-inflammatory activity of URB1537 128 µg/mL was comparable to that of the reference drug dexamethasone 2 µg/mL, suggesting that this sugar ester might help to control the inflammatory response. NO release was not increased in non-stimulated RAW 264.7 cells after URB1537 administration, indicating that the compound did not lead to immune system activation (data not shown).

Overall, these data agree with previous observations demonstrating that sucrose fatty acid esters exhibit anti-inflammatory effects through different mechanisms, such as the inhibition of NF-κB activation involved in immunity and inflammation [41]. Similarly, we have recently observed that some lactose-based fatty acid esters may counteract NO production in LPS-stimulated macrophages [24].

**Figure 4:**
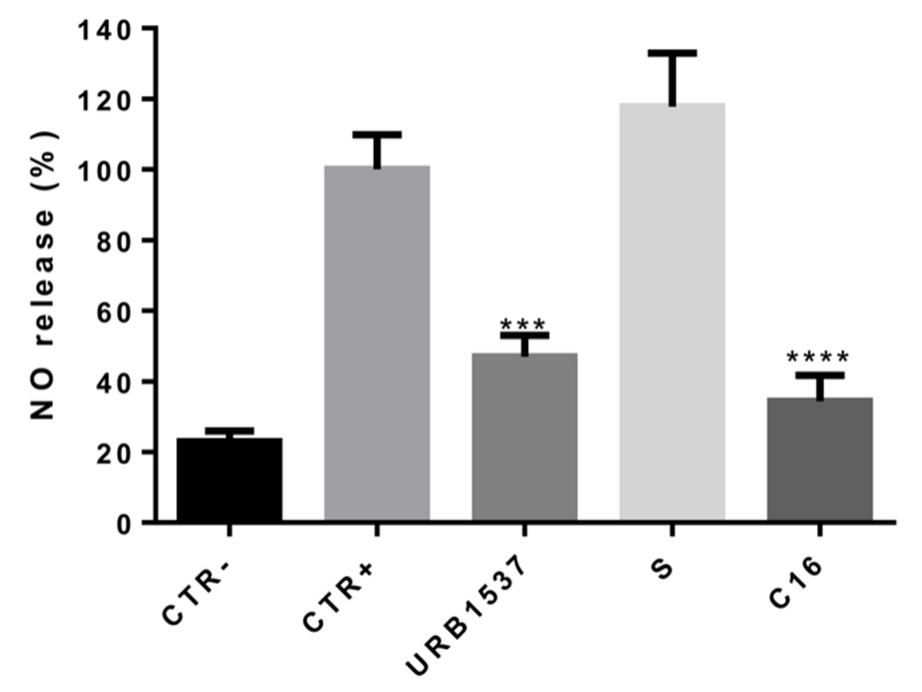
Extracellular NO release after RAW 264.7 stimulation by LPS for 24 h in the presence of URB1537, S, and C16 (128 µg/mL). CTR-: negative control (untreated cells); CTR+: positive control (LPS). Data are expressed as the mean ± SD (*n* = 3). *** *p* < 0.001, **** *p* < 0.0001 vs. CTR+ (Tukey’s post hoc test).

The anti-inflammatory properties of URB1537 were also compared to those of its single constituents, i.e., sucrose (S) and palmitoleic acid (C16). As indicated in Figure 4, URB1537 and C16 presented a similar anti-inflammatory activity (*p* = ns URB1537 vs. C16), significantly reducing LPS-induced NO production. Sucrose alone did not show anti-inflammatory properties. This finding suggests that the anti-inflammatory capacity of URB1537 could be linked to the presence of the palmitoleate portion. Accordingly, it has been previously demonstrated that palmitoleic acid promotes anti-inflammatory effects in macrophages exposed to LPS by inhibiting the inflammasome pathway [42]. However, its real application could be limited by its oily nature, low solubility, and tendency to irritate (Safety Data Sheet). Indeed, URB1537 could be considered a safe potential prodrug of palmitoleic acid with better physicochemical and pharmacokinetic properties.

**Figure 5:**
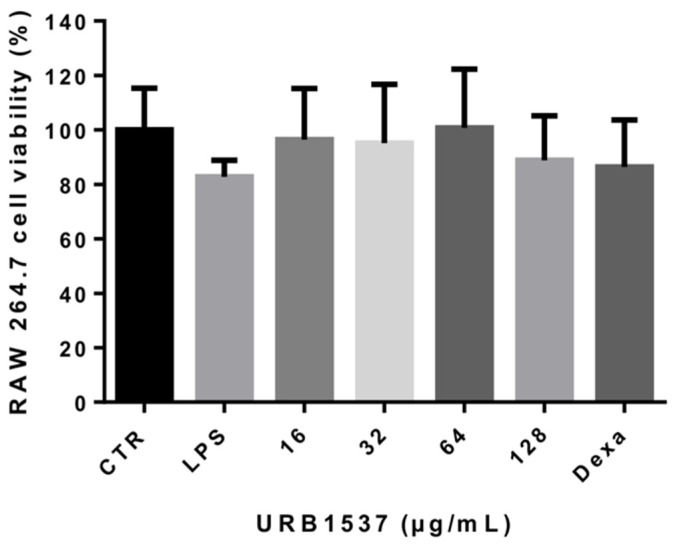
Cell viability evaluation after LPS (1 µg/mL), URB1537 (16–128 µg/mL), and dexamethasone (2 µg/mL) administration to RAW 264.7 cells for 24 h. Data are expressed as the mean ± SD (*n* = 3).

Noteworthily, no cytotoxic effects were observed after LPS, URB1537, and dexamethasone administration to RAW 264.7 cells compared to untreated control cells (Figure 5), confirming that URB1537 was safe at concentrations up to 128 µg/mL, as also evidenced on HaCaT cells.

## 3. Materials and Methods

### 3.1. Chemicals

Caprylic and undecynoic acids were purchased from TCI (Zwijndrecht, Belgium). Undecylenic, palmitoleic, oleic, linoleic, linolenic, and nervonic acids, and sucrose were purchased from Fluorochem (Hadfield, UK). Diisopropyl azodicarboxylate (DIAD) and triphenylphosphine (PPh_3_) were purchased from Alpha Aesar (Ward Hill, MA, USA). Benzoic, phenylacetic, phenylbenzoic, and biphenylacetic acids, dichloromethane (CH_2_Cl_2_), acetone [CH_3_C(O)CH_3_], methanol (CH_3_OH), dimethyl sulfoxide (DMSO and DMSO-*d6*) were purchased from Sigma-Aldrich (Milan, Italy). The structures of compounds were unambiguously assessed by MS, ^1^H NMR, ^13^C NMR, and IR. ESI-MS spectra were recorded with a Waters Micromass ZQ spectrometer in a negative or positive mode using nebulizing nitrogen gas at 400 L/min and a temperature of 250 °C, cone flow 40 mL/min, capillary 3.5 kV and cone voltage 60 V; only molecular ions [M-H]^−^ or [M + NH_4_]^+^ or [M + Na]^+^ are given. HRMS spectra were performed by slow direct infusion (5 μL/min) of ≈ 0.1 μg/mL solution (acetonitrile/0.1% aqueous formic acid 1:1) of new compounds, using Orbitrap Exploris 240 mass spectrometer (Thermo Scientific, Waltham, MA, USA); only molecular ions [M + Na]^+^ are given. ^1^H NMR and ^13^C NMR spectra were recorded on a Bruker AC 400 or 101, respectively, spectrometer and analyzed using the TopSpin 1.3 software package. Chemical shifts were measured by using the central peak of the solvent. Column chromatography purifications were performed under “flash” conditions using Merck 230–400 mesh silica gel. TLC was carried out on Merck silica gel 60 F254 plates, which were visualized by exposure to ultraviolet light and by exposure to an aqueous solution of ceric ammonium molybdate.

### 3.2. General Procedure for the Synthesis of Sucrose Ester Surfactants (**3a–m**, URB1480-1482, URB1534-1543)

Sucrose (**1**) (0.342 g, 1 mmol) was dissolved in dry DMF (7.9 mL) at 70 °C and stirred under N_2_ atmosphere. The mixture was cooled to room temperature and then PPh_3_ (0.656 g, 2.5 mmol), the appropriate carboxylic acid (**2a–m**) (1.5 mmol), and DMF (2.1 mL) were added. After complete dissolution, the mixture was cooled to 0 °C and DIAD (0.508 g, 0.493 mL, 2.5 mmol) was introduced. The mixture was stirred at room temperature until the total consumption of sucrose (24–30 h) and concentrated. Purification of the residue by column chromatography (CH_2_Cl_2_/CH_3_C(O)CH_3_/MeOH/H_2_O 78:10:10:1.5) gave **3a–m** as solids.

β-d-Fructofuranosyl 6-*O*-octanoyl-α-d-glucopyranoside, sucrose caprylate (**3a**, URB1534) [43].

White solid. Yield: 50%. MS (ESI): 467 [M–H]^−^, 486 [M + NH_4_]^+^, 491 [M + Na]^+^. ^1^H NMR (DMSO-*d*_6_): δ = 0.86 (t, 3H, *J* = 7.0 Hz, CH_3_) 1.22–1.30 [m, 8H, (CH_2_)_4_], 1.48–1.55 (m, 2H, C*H*_2_CH_2_COOR), 2.29–2.33 (m, 2H, C*H*_2_COOR), 3.06 (ddd, 1H, *J*_H4-OH4_ = 6.0 Hz, *J*_H4-H5_ = 9.0 Hz, *J*_H4-H3_ = 9.5 Hz, H4), 3.21 (ddd, 1H, *J*_H2-H1_ = 3.5 Hz, *J*_H2-OH2_ = 6.0 Hz, *J*_H2-H3_ = 9.5 Hz, H2), 3.38–3.41 (m, 2H, H1′a, H1′b), 3.49 (ddd, 1H, *J*_H3-OH3_ = 5.0 Hz, *J*_H3-H2_ ≈ *J*_H3-H4_ = 9.5 Hz, H3), 3.53–3.60 (m, 3H, H5′, H6′a, H6′b), 3.70–3.76 (m, 1H, H4′), 3.88 (dd, 1H, *J*_H3′-OH3′_ ≈ *J*_H3′-H4′_= 8.0 Hz, H3′), 3.90 (ddd, 1H, *J*_H5-H6b_ = 1.5 Hz, *J*_H5-H6a_ = 6.0 Hz, *J*_H5-H4_ = 9.0 Hz, H5), 4.02 (dd, 1H, *J*_H6a-H5_ = 6.0 Hz, *J*_H6a-H6b_ = 11.5 Hz, H6a), 4.23 (dd, 1H, *J*_H6b-H5_ = 1.5 Hz, *J*_H6b-H6a_ = 11.5 Hz, H6b), 4.38 (dd, 1H, *J*_OH6′-H6′a_ ≈ *J*_OH6′-H6′b_ = 6.0 Hz, OH6′), 4.55 (d, 1H, *J*_OH3′-H3′_ = 8.0 Hz, OH3′), 4.80 (dd, 1H, *J*_OH1′-H1′a_ ≈ *J*_OH1′-H1′b_ = 6.5 Hz, OH1′), 4.88 (d, 1H, *J*_OH3-H3_ = 5.0 Hz, OH3), 5.00 (d, 1H, *J*_OH4-H4_ = 6.0 Hz, OH4), 5.12 (d, 1H, *J*_OH2-H2_ = 6.0 Hz, OH2), 5.15 (d, 1H, *J*_OH4′-H4′_ = 6.0 Hz, OH4′), 5.18 (d, 1H, *J*_H1-H2_ = 3.5 Hz, H1) ppm. ^13^C NMR (DMSO-*d*_6_): δ = 14.4, 22.5, 24.9, 28.8, 28.9, 31.6, 33.8, 62.6, 63.1, 64.0, 70.4, 70.6, 72.0, 73.1, 75.0, 77.3, 83.2, 91.9, 104.3, 173.5 ppm.

β-d-Fructofuranosyl 6-*O*-undec-10-enoyl-α-d-glucopyranoside, sucrose undec-10-enoate (**3b**, URB1535).

White solid. Yield: 39%. MS (ESI): 507 [M–H]^−^, 526 [M + NH_4_]^+^, 531 [M + Na]^+^. ^1^H NMR (DMSO-*d*_6_): δ = 1.24–1.27 [m, 8H, (CH_2_)_4_], 1.33–1.35 (m, 2H, C*H*_2_CH_2_CH=CH_2_), 1.49–1.53 (m, 2H, C*H*_2_CH_2_COOR), 1.98–2.04 (m, 2H, C*H*_2_CH=CH_2_), 2.28–2.32 (m, 2H, *CH*_2_COOR), 3.06 (ddd, 1H, *J*_H4-OH4_ = 5.0 Hz, *J*_H4_-_H3_ ≈ *J*_H4-H5_ = 9.5 Hz, H4), 3.20 (ddd, 1H, *J*_H2-H1_ = 3.5 Hz, *J*_H2-OH2_ = 6.0 Hz, *J*_H2-H3_ = 9.5 Hz, H2), 3.37–3.40 (m, 2H, H1′a, H1′b), 3.48 (m, 1H, H3), 3.53–3.62 (m, 3H, H5′, H6′a, H6′b), 3.70–3.76 (m, 1H, H4′), 3.88 (dd, 1H, *J*_H3′-OH3′_ ≈ *J*_H3′-H4′_= 8.0 Hz, H3′), 3.91 (ddd, 1H, *J*_H5-H6b_ = 1.5 Hz, *J*_H5-H6a_ = 6.0 Hz, *J*_H5-H4_ = 9.5 Hz, H5), 4.01 (dd, 1H, *J*_H6a-H5_ = 6.0 Hz, *J*_H6a-H6b_ = 11.5 Hz, H6a), 4.23 (dd, 1H, *J*_H6b-H5_ = 1.5 Hz, *J*_H6b-H6a_ = 11.5 Hz, H6b), 4.39 (dd, 1H, *J*_OH6′-H6′a_ ≈ *J*_OH6′-H6′b_ = 5.0 Hz, OH6′), 4.56 (d, 1H, *J*_OH3′-H3′_ = 8.0 Hz, OH3′), 4.81 (dd, 1H, *J*_OH1′-H1′a_ ≈ *J*_OH1′-H1′b_ = 6.0 Hz, OH1′), 4.89 (brs, 1H, OH3), 4.94 (dddd, 1H, *J*_gem_ ≈ *J*_1_ = 1.5 Hz, *J*_2_ = 4.0 Hz, *J*_cis_ = 10.0 Hz, HC*H*=CHCH_2_), 5.00 (dddd, 1H, *J*_gem_ ≈ *J*_1_ ≈ *J*_2_ = 1.5 Hz, *J*_trans_ = 17.0 Hz, *H*CH=CHCH_2_), 5.01 (d, 1H, *J*_OH4-H4_ = 5.0 Hz, OH4), 5.13 (d,1H, *J*_OH2-H2_ = 6.0 Hz, OH2), 5.16 (d, 1H, *J*_OH4′-H4′_ = 6.0 Hz, OH4′), 5.18 (d, 1H, *J*_H1-H2_ = 3.5 Hz, H1), 5.80 (dddd, 1H, *J*_1_ ≈ *J*_2_ = 7.0 Hz, *J*_cis_ = 10.0 Hz, *J*_trans_ = 17.0 Hz, CH_2_=C*H*CH_2_) ppm. ^13^C NMR (DMSO-*d*_6_): δ = 24.9, 28.7, 28.9, 29.1, 29.1, 29.2, 33.6, 33.8, 62.6, 63.1, 64.0, 70.5, 70.6, 72.0, 73.1, 75.0, 77.4, 83.2, 91.9, 104.4, 115.1, 139.3, 173.5 ppm. HRMS (ESI) m/z for C_23_H_40_NaO_12_ [M + Na]^+^: calcd 531.2412; found 531.2411.

β-d-Fructofuranosyl 6-*O*-undec-10-inoyl-α-d-glucopyranoside, sucrose undec-10-inoate (**3c**, URB1536).

White solid. Yield: 37%. MS (ESI): 505 [M–H]^−^, 524 [M + NH_4_]^+^, 529 [M + Na]^+^. ^1^H NMR (DMSO-*d*_6_): δ = 1.23–1.27 [m, 6H, (CH_2_)_3_], 1.30–1.37 (m, 2H, *CH*_2_CH_2_CH_2_CCH), 1.40–1.47 (m, 2H, *CH*_2_CH_2_CCH), 1.49–1.55 (m, 2H, C*H*_2_CH_2_COOR), 2.14 (td, 2H, *J*_1_ = 2.5 Hz, *J*_2_ = 7.0 Hz, C*H*_2_CCH), 2.28–2.33 (m, 2H, *CH*_2_COOR), 2.72 (t, 1H, *J* = 2.5 Hz, C*CH*), 3.06 (ddd, 1H, *J*_H4-OH4_ = 5.0 Hz, *J*_H4_-_H3_ ≈ *J*_H4-H5_ = 9.5 Hz, H4), 3.21 (ddd, 1H, *J*_H2-H1_ = 3.5 Hz, *J*_H2-OH2_ = 6.0 Hz, *J*_H2-H3_ = 9.5 Hz, H2), 3.36–3.41 (m, 2H, H1′a, H1′b), 3.49 (ddd, 1H, *J*_H3-OH3_ = 5.0 Hz, *J*_H3-H2_ ≈ *J*_H3-H4_ = 9.5 Hz, H3), 3.53–3.62 (m, 3H, H5′, H6′a, H6′b), 3.70–3.76 (m, 1H, H4′), 3.88 (dd, 1H, *J*_H3′-OH3′_ ≈ *J*_H3′-H4′_ = 8.0 Hz, H3′), 3.90 (ddd, 1H, *J*_H5-H6b_ = 1.5 Hz, *J*_H5-H6a_ = 6.0 Hz, *J*_H5-H4_ = 9.5 Hz, H5), 4.02 (dd, 1H, *J*_H6a-H5_ = 6.0 Hz, *J*_H6a-H6b_ = 11.5 Hz, H6a), 4.23 (dd, 1H, *J*_H6b-H5_ = 1.5 Hz, *J*_H6b-H6a_ = 11.5 Hz, H6b), 4.38 (dd, 1H, *J*_OH6′-H6′a_ ≈ *J*_OH6′-H6′b_ = 5.0 Hz, OH6′), 4.56 (d, 1H, *J*_OH3′-H3′_ = 8.0 Hz, OH3′), 4.80 (dd, 1H, *J*_OH1′-H1′a_ ≈ *J*_OH1′-H1′b_ = 6.0 Hz, OH1′), 4.88 (d, 1H, *J*_OH3-H3_ = 5.0 Hz, OH3), 5.00 (d, 1H, *J*_OH4-H4_ = 5.0 Hz, OH4), 5.13 (d, 1H, *J*_OH2-H2_ = 6.0 Hz, OH2), 5.16 (d, 1H, *J*_OH4′-H4′_ = 6.0 Hz, OH4′), 5.18 (d, 1H, *J*_H1-H2_ = 3.5 Hz, H1) ppm. ^13^C NMR (DMSO-*d*_6_) δ 18.1, 24.9, 28.4, 28.5, 28.8, 28.9, 29.1, 33.8, 62.7, 63.0, 64.0, 70.5, 70.6, 71.5, 72.0, 73.1, 75.0, 77.4, 83.2, 85.0, 91.9, 104.4, 173.5 ppm. HRMS (ESI) m/z for C_23_H_38_NaO_12_ [M + Na]^+^: calcd 529.2256; found 529.2253.

β-d-Fructofuranosyl 6-*O*-hexadec-9-enoyl-α-d-glucopyranoside, sucrose palmitoleate (**3d**, URB1537) [25].

White solid. Yield: 30%. MS (ESI): 577 [M–H]^−^, 596 [M + NH_4_]^+^, 601 [M + Na]^+^. ^1^H NMR (DMSO-*d*_6_): δ = 0.86 (t, 3H, *J* = 7.0 Hz, CH_3_), 1.26–1.30 [m, 16H, (CH_2_)_8_], 1.50–1.53 (m 2H, C*H*_2_CH_2_COOR), 1.97–2.00 (m, 4H, C*H*_2_CH=CHC*H*_2_), 2.28–2.32 (m, 2H, C*H*_2_COOR), 3.06 (ddd, 1H, *J*_H4-OH4_ = 5.0 Hz, *J*_H4-H3_ ≈ *J*_H4-H5_ = 9.5 Hz, H4), 3.19 (ddd, 1H, *J*_H2-H1_ = 3.5 Hz, *J*_H2-OH2_ = 4.0 Hz, *J*_H2-H3_ = 9.5 Hz, H2), 3.38–3.39 (m, 2H, H1′a, H1′b), 3.49 (m, 1H, H3), 3.54–3.62 (m, 3H, H5′, H6′a, H6′b), 3.71–3.76 (m, 1H, H4′), 3.88 (dd, 1H, *J*_H3′-OH3′_ ≈ *J*_H3′-H4′_ = 8.0 Hz, H3′), 3.91 (ddd, 1H, *J*_H5-H6b_ = 1.5 Hz, *J*_H5-H6a_ = 6.0 Hz, *J*_H5-H4_ = 9.5 Hz, H5), 4.03 (dd, 1H, *J*_H6a-H5_ = 6.0 Hz, *J*_H6a-H6b_ = 11.5 Hz, H6a), 4.23 (dd, 1H, *J*_H6b-H5_ = 1.5 Hz, *J*_H6b-H6a_ = 11.5 Hz, H6b), 4.38 (dd, 1H, *J*_OH6′-H6′a_ ≈ *J*_OH6′-H6′b_ = 5.0 Hz, OH6′), 4.55 (d, 1H, *J*_OH3′-H3′_ = 8.0 Hz, OH3′), 4.80 (dd, 1H, *J*_OH1′-H1′a_ ≈ *J*_OH1′-H1′b_ = 6.0 Hz, OH1′), 4.88 (brs, 1H, OH3), 5.00 (d, 1H, *J*_OH4-H4_ = 5.0 Hz, OH4), 5.12 (d, 1H, *J*_OH2-H2_ = 4.0 Hz, OH2), 5.14 (d, 1H, *J*_OH4′-H4′_ = 6.0 Hz, OH4′), 5.18 (d, 1H, *J*_H1-H2_ = 3.5 Hz, H1), 5.31 (ddd, 1H, *J*_1_ ≈ *J*_2_ = 6.0 Hz, *J*_3_ = 11.0 Hz, CH=CH), 5.34 (ddd, 1H, *J*_1_ ≈ *J*_2_ = 6.0 Hz, *J*_3_ = 11.0 Hz, CH=CH) ppm. ^13^C NMR (DMSO-*d*_6_): δ = 14.4, 22.5, 24.8, 27.1 (2C), 28.7, 29.0, 29.1, 29.6, 31.6, 33.8, 62.6, 63.1, 64.0, 70.4, 70.6, 72.0, 73.1, 75.0, 77.4, 83.2, 91.9, 104.4, 130.1 (2C), 173.5 ppm.

β-d-Fructofuranosyl 6-*O*-octadec-9-enoyl-α-d-glucopyranoside, sucrose oleate (**3e**, URB1538) [44].

White solid. Yield: 36%. MS (ESI): 605 [M–H]^−^, 624 [M + NH_4_]^+^, 629 [M + Na]^+^. ^1^H NMR (DMSO-*d*_6_): δ = 0.86 (t, 3H, *J* = 6.5 Hz, CH_3_) 1.22–1.33 [m, 20H, (CH_2_)_10_], 1.47–1.55 (m, 2H, C*H*_2_CH_2_COOR), 1.95–2.01 (m, 4H, C*H*_2_CH=CHC*H*_2_), 2.28–2.32 (m, 2H, C*H*_2_COOR), 3.06 (ddd, 1H, *J*_H4-OH4_ = 6.0 Hz, *J*_H4-H3_ ≈ *J*_H4-H5_ = 9.5 Hz, H4), 3.20 (ddd, 1H, *J*_H2-H1_ = 3.5 Hz, *J*_H2-OH2_ = 6.0 Hz, *J*_H2-H3_ = 9.5 Hz, H2), 3.39–3.40 (m, 2H, H1′a, H1′b), 3.48 (ddd, 1H, *J*_H3-OH3_ = 5.0 Hz, *J*_H3-H2_ ≈ *J*_H3-H4_ = 9.5 Hz, H3), 3.52–3.62 (m, 3H, H5′, H6′a, H6′b), 3.70–3.76 (m, 1H, H4′), 3.87 (dd, 1H, *J*_H3′-OH3′_ ≈ *J*_H3′-H4′_= 8.0 Hz, H3′), 3.91 (ddd, 1H, *J*_H5-H6b_ = 1.5 Hz, *J*_H5-H6a_ = 6.0 Hz, *J*_H5-H4_ = 9.5 Hz, H5), 4.02 (dd, 1H, *J*_H6a-H5_ = 6.0 Hz, *J*_H6a-H6b_ = 11.5 Hz, H6a), 4.23 (dd, 1H, *J*_H6b-H5_ = 1.5 Hz, *J*_H6b-H6a_ = 11.5 Hz, H6b), 4.39 (dd, 1H, *J*_OH6′-H6′a_ ≈ *J*_OH6′-H6′b_ = 5.0 Hz, OH6′), 4.56 (d, 1H, *J*_OH3′-H3′_ = 8.0 Hz, OH3′), 4.81 (dd, 1H, *J*_OH1′-H1′a_ ≈ *J*_OH1′-H1′b_ = 6.0 Hz, OH1′), 4.88 (d, 1H, *J*_OH3-H3_ = 5.0 Hz, OH3), 5.00 (d, 1H, *J*_OH4-H4_ = 6.0 Hz, OH4), 5.13 (d, 1H, *J*_OH2-H2_ = 6.0 Hz, OH2), 5.16 (d, 1H, *J*_OH4′-H4′_ = 6.0 Hz, OH4′), 5.18 (d, 1H, *J*_H1-H2_ = 3.5 Hz, H1), 5.31 (ddd, 1H, *J*_1_ ≈ *J*_2_ = 6.0 Hz, *J*_3_ = 11.0 Hz, CH=CH), 5.35 (ddd, 1H, *J*_1_ ≈ *J*_2_ = 6.0 Hz, *J*_3_ = 11.0 Hz, CH=CH) ppm. ^13^C NMR (DMSO-*d*_6_): δ = 14.4, 22.5, 24.9, 27.0, 27.1, 28.95, 28.97, 29.03, 29.06, 29.12, 29.3, 29.6, 31.7, 33.7, 62.6, 63.0, 64.0, 70.4, 70.6, 72.0, 73.1, 75.0, 77.4, 83.2, 92.0, 104.4, 130.1(2C), 173.5 ppm.

β-d-Fructofuranosyl 6-*O*-octadec-9,12-enoyl-α-d-glucopyranoside, sucrose linoleate (**3f**, URB1539) [41].

White solid. Yield: 23%. MS (ESI): 603 [M–H]^−^, 622 [M + NH_4_]^+^, 627 [M + Na]^+^. ^1^H NMR (DMSO-*d*_6_): δ = 0.86 (t, 3H, *J* = 6.5 Hz, CH_3_), 1.22–1.34 [m, 14H, (CH_2_)_7_], 1.49–1.54 (m 2H, C*H*_2_CH_2_COOR), 2.00–2.05 (m, 4H, C*H*_2_CH=CHCH_2_CH=CHC*H*_2_), 2.28–2.32 (m, 2H, C*H*_2_COOR), 2.74 (m, 2H, CH=CHC*H*_2_CH=CH), 3.06 (ddd, 1H, *J*_H4-OH4_ = 5.5 Hz, *J*_H4-H3_≈*J*_H4-H5_ = 9.0 Hz, H4), 3.20 (ddd, 1H, *J*_H2-H1_ = 3.5 Hz, *J*_H2-OH2_ = 6.0 Hz, *J*_H2-H3_ = 9.0 Hz, H2), 3.39–3.40 (m, 2H, H1′a, H1′b), 3.48 (m, 1H, H3), 3.53–3.61 (m, 3H, H5′, H6′a, H6′b), 3.71–3.77 (m, 1H, H4′), 3.87 (dd, 1H, *J*_H3′-OH3′_ ≈ *J*_H3′-H4′_ = 8.0 Hz, H3′), 3.90–3.93 (m, 1H, H5), 4.02 (dd, 1H, *J*_H6a-H5_ = 6.0 Hz, *J*_H6a-H6b_ = 11.5 Hz, H6a), 4.22 (dd, 1H, *J*_H6b-H6a_ = 11.5 Hz, H6b), 4.38 (dd, 1H, *J*_OH6′-H6′a_ ≈ *J*_OH6′-H6′b_ = 5.0 Hz, OH6′), 4.56 (d, 1H, *J*_OH3′-H3′_ = 8.0 Hz, OH3′), 4.81 (dd, 1H, *J*_OH1′-H1′a_ ≈ *J*_OH1′-H1′b_ = 6.0 Hz, OH1′), 4.89 (d, 1H, *J*_OH3-H3_ = 4.5 Hz, OH3), 5.00 (d, 1H, *J*_OH4-H4_ = 5.5 Hz, OH4), 5.12 (d,1H, *J*_OH2-H2_ = 6.0 Hz, OH2), 5.14 (d, 1H, *J*_OH4′-H4′_ = 6.5 Hz, OH4′), 5.18 (d, 1H, *J*_H1-H2_ = 3.5 Hz, H1), 5.27–5.38 (m, 4H, C*H*=C*H*CH_2_C*H*=C*H*) ppm. ^13^C NMR (DMSO-*d*_6_): δ = 14.4, 22.4, 24.9, 25.7, 27.0, 27.1, 28.95, 29.0, 29.1, 29.2, 29.5, 31.3, 33.8, 62.6, 63.0, 64.0, 70.4, 70.6, 72.0, 73.0, 75.0, 77.4, 83.2, 91.9, 104.4, 128.2(2C), 130.2(2C), 173.5 ppm.

β-d-Fructofuranosyl 6-*O*-octadec-9,12,15-enoyl-α-d-glucopyranoside, sucrose linolenate (**3g**, URB1540).

White solid. Yield: 25%. MS (ESI): 601 [M–H]^–^, 620 [M + NH_4_]^+^, 625 [M + Na]^+^. ^1^H NMR (DMSO-*d*_6_): δ = 0.93 (t, 3H, *J* = 7.5 Hz, CH_3_) 1.19–1.26 [m, 8H, (CH_2_)_4_], 1.49–1.54 (m 2H, C*H*_2_CH_2_COOR), 2.02–2.08 (m, 4H, C*H*_2_CH=CHCH_2_CH=CHCH_2_CH=CHC*H*_2_), 2.28–2.32 (m, 2H, C*H*_2_COOR), 2.73–2.79 (m, 4H, CH=CHC*H*_2_CH=CHC*H*_2_CH=CH), 3.06 (ddd, 1H, *J*_H4-OH4_ = 6.0 Hz, *J*_H4-H3_ ≈ *J*_H4-H5_ = 9.5 Hz, H4), 3.21 (ddd, 1H, *J*_H2-H1_ = 3.5 Hz, *J*_H2-OH2_ = 6.0 Hz, *J*_H2-H3_ = 9.5 Hz, H2), 3.38–3.39 (m, 2H, H1′a, H1′b), 3.49 (ddd, 1H, *J*_H3-OH3_ = 5.0 Hz, *J*_H3-H2_ ≈ *J*_H3-H4_ = 9.5 Hz, H3), 3.54–3.61 (m, 3H, H5′, H6′a, H6′b), 3.70–3.76 (m, 1H, H4′), 3.86 (dd, 1H, *J*_H3′-OH3′_ ≈ *J*_H3′-H4′_ = 8.0 Hz, H3′), 3.90–3.93 (m, 1H, H5), 4.02 (dd, 1H, *J*_H6a-H5_ = 6.0 Hz, *J*_H6a-H6b_ = 11.5 Hz, H6a), 4.23 (dd, 1H, *J*_H6b-H5_ = 1.5 Hz, *J*_H6b-H6a_ = 11.5 Hz, H6b), 4.38 (dd, 1H, *J*_OH6′-H6′a_ ≈ *J*_OH6′-H6′b_ = 5.0 Hz, OH6′), 4.56 (d, 1H, *J*_OH3′-H3′_ = 8.0 Hz, OH3′), 4.80 (dd, 1H, *J*_OH1′-H1′a_ ≈ *J*_OH1′-H1′b_ = 6.5 Hz, OH1′), 4.89 (d, 1H, *J*_OH3-H3_ = 5.0 Hz, OH3), 5.00 (d, 1H, *J*_OH4-H4_ = 6.00 Hz, OH4), 5.13 (d, 1H, *J*_OH2-H2_ = 6.0 Hz, OH2), 5.15 (d, 1H, *J*_OH4′-H4′_ = 6.0 Hz, OH4′), 5.18 (d, 1H, *J*_H1-H2_ = 3.5 Hz, H1), 5.25–5.40 (m, 6H, C*H*=C*H*CH_2_C*H*=C*H*CH_2_C*H*=C*H*) ppm. ^13^C NMR (DMSO-*d*_6_): δ = 14.6, 20.5, 24.8, 25.6, 25.7, 27.1, 28.9, 29.0, 29.1, 29.5, 33.8, 62.6, 63.0, 64.0, 70.4, 70.6, 72.0, 73.1, 75.0, 77.4, 83.2, 92.0, 104.4, 127.4, 128.0, 128.3, 128.4, 130.4, 132.0, 173.5 ppm. HRMS (ESI) m/z for C_30_H_50_NaO_12_ [M + Na]^+^: calcd 625.3195; found 625.3191.

β-d-Fructofuranosyl 6-*O*-tetracos-15-enoyl-α-d-glucopyranoside, sucrose nervonate (**3h**, URB1541).

White solid. Yield: 26%. MS (ESI): 689 [M–H]^−^ 708 [M + NH_4_]^+^, 713 [M + Na]^+^. ^1^H NMR (DMSO-*d*_6_): δ = 0.86 (t, 3H, *J* = 7.0 Hz, CH_3_), 1.24–1.29 [m, 32H, (CH_2_)_16_], 1.49–1.53 (m 2H, C*H*_2_CH_2_COOR), 1.96–2.00 (m, 4H, C*H*_2_CH=CHC*H*_2_), 2.30 (t, 2H, *J* = 7.5 Hz, C*H*_2_COOR), 3.06 (ddd, 1H, *J*_H4-OH4_ = 5.5 Hz, *J*_H4_-_H3_ ≈ *J*_H4-H5_ = 9.5 Hz, H4), 3.21 (ddd, 1H, *J*_H2-H1_ = 3.5 Hz, *J*_H2-OH2_ = 6.0 Hz, *J*_H2-H3_ = 9.5 Hz, H2), 3.38–3.40 (m, 2H, H1′a, H1′b), 3.49 (dd, 1H, *J*_H3-H2_ ≈ *J*_H3-H4_ = 9.5 Hz, H3), 3.54–3.62 (m, 3H, H5′, H6′a, H6′b), 3.71–3.76 (m, 1H, H4′), 3.88 (dd, 1H, *J*_H3′-OH3′_ ≈ *J*_H3′-H4′_ = 8.0 Hz, H3′), 3.89–3.93 (m, 1H, H5), 4.02 (dd, 1H, *J*_H6a-H5_ = 6.0 Hz, *J*_H6a-H6b_ = 11.5 Hz, H6a), 4.23 (dd, 1H, *J*_H6b-H5_ = 1.5 Hz, *J*_H6b-H6a_ = 11.5 Hz, H6b), 4.37 (dd, 1H, *J*_OH6′-H6′a_ ≈ *J*_OH6′-H6′b_ = 5.5 Hz, OH6′), 4.56 (d, 1H, *J*_OH3′-H3′_ = 8.0 Hz, OH3′), 4.80 (dd, 1H, *J*_OH1′-H1′a_ ≈ *J*_OH1′-H1′b_ = 6.5 Hz, OH1′), 4.89 (d, 1H, *J*_OH3-H3_ = 3.5 Hz, OH3), 5.00 (d, 1H, *J*_OH4-H4_ = 5.5 Hz, OH4), 5.13 (d, 1H, *J*_OH2-H2_ = 6.0 Hz, OH2), 5.15 (d, 1H, *J*_OH4′-H4′_ = 6.0 Hz, OH4′), 5.18 (d, 1H, *J*_H1-H2_ = 3.5 Hz, H1), 5.31 (ddd, 1H, *J*_1_ ≈ *J*_2_ = 6.0 Hz, *J*_3_ = 11.0 Hz, CH=CH), 5.34 (ddd, 1H, *J*_1_ ≈ *J*_2_ = 6.0 Hz, *J*_3_ = 11.0 Hz CH=CH) ppm ^13^C NMR (DMSO-*d*_6_): δ = 14.3, 22.6, 24.9, 27.0, 29.0, 29.1, 29.29, 29.31, 29.4, 29.5, 31.75, 33.8, 62.7, 63.0, 64.0, 70.5, 70.6, 72.0, 73.1, 75.0, 77.4, 83.2, 92.0, 104.4, 130.1, 173.5 ppm. HRMS (ESI) m/z for C_36_H_66_NaO_12_ [M + Na]^+^: calcd 713.4447; found 713.4443.

β-d-Fructofuranosyl 6-*O*-benzoyl-α-d-glucopyranoside, sucrose benzoate (**3i**, URB1542) [45].

White solid. Yield: 43%. MS (ESI): 445 [M–H]^−^, 428 [M + NH_4_]^+^, 433 [M + Na]^+^. ^1^H NMR (DMSO-*d*_6_): δ = 3.26 (ddd, 1H, *J*_H4-OH4_ = 6.0 Hz, *J*_H4-H3_ ≈ *J*_H4-H5_ = 9.5 Hz, H4), 3.28 (ddd, 1H, *J*_H2-H1_ = 3.5 Hz, *J*_H2-OH2_ = 6.0 Hz, *J*_H2-H3_ = 9.5 Hz, H2), 3.41–3.43 (m, 2H, H1′a, H1′b), 3.50–3.61 (m, 4H, H3, H5′, H6′a, H6′b), 3.79 (ddd, 1H, *J*_H4′-OH4_ = 6.00 Hz, *J*_H4′-H3′_ ≈ *J*_H4′-H5′_ = 8.00 Hz, H4′), 3.91 (dd, 1H, *J*_H3′-OH3′_ ≈ *J*_H3′-H4′_ = 8.0 Hz, H3′), 4.04–4.11 (m, 1H, H5), 4.34 (dd, 1H, *J*_H6a-H5_ = 5.0 Hz, *J*_H6a-H6b_ = 11.5 Hz, H6a), 4.37 (dd, 1H, *J*_OH6′-H6′a_ ≈ *J*_OH6′-H6′b_ = 5.5 Hz, OH6′), 4.45 (dd, 1H, *J*_H6b-H5_ = 1.5 Hz, *J*_H6b-H6a_ = 11.5 Hz, H6b), 4.65 (d, 1H, *J*_OH3′-H3′_ = 8.0 Hz, OH3′), 4.83 (dd, 1H, *J*_OH1′-H1′a_ ≈ *J*_OH1′-H1′b_ = 6.0 Hz, OH1′), 4.92 (d, 1H, *J*_OH3-H3_ = 5.0 Hz, OH3), 5.14 (d, 1H, *J*_OH4-H4_ = 6.0 Hz, OH4), 5.15 (d, 1H, *J*_OH2-H2_ = 6.0 Hz, OH2), 5.19 (d, 1H, *J*_OH4′-H4′_ = 6.0 Hz, OH4′), 5.23 (d, 1H, *J*_H1-H2_ = 3.5 Hz, H1), 7.51–7.57 (m, 2H, ArH), 7.67 [dddd, 1H, *J*_1_ ≈ *J*_2_ = 1.0 Hz, *J*_3_ ≈ *J*_4_ = 8.5 Hz, ArH(*p)*], 7.97–8.01 (m, 2H, ArH) ppm. ^13^C NMR (DMSO-*d*_6_): δ = 62.6, 63.0, 64.8, 70.5, 72.0, 73.1, 75.0, 77.4, 83.1, 92.2, 104.5, 129.2, 129.7, 130.1, 133.7, 173.5 ppm.

β-d-Fructofuranosyl 6-*O*-[2-(4-phenyl)benzoyl]-α-d-glucopyranoside, sucrose *p*-phenyl benzoate (**3j**, URB1481).

Pale yellow solid. Yield: 34%. MS (ESI): 521 [M-H]^−^, 540 [M + NH_4_]^+^, 545 [M + Na]^+^. ^1^H NMR (DMSO-*d*_6_): δ = 3.25–3.32 (m, 2H, H^4^, H^2^), 3.40–3.44 (m, 2H, H^1′a^, H^1′b^), 3.48 (ddd, 1H, *J*_H3-OH3_ = 5.0 Hz, *J*_H3-H2_ ≈ *J*_H3-H4_ = 9.0 Hz, H^3^), 3.52–3.62 (m, 3H, H^5′^, H^6′a^, H^6′b^), 3.78–3.83 (m, 1H, H^4′^), 3.92 (dd, 1H, *J*_H3′-OH3′_ ≈ *J*_H3′-H4′_ = 8.0 Hz, H^3′^), 4.09 (ddd, 1H, *J*_H5-H6b_ = 1.5 Hz, *J*_H5-H6a_ = 5.0 Hz, *J*_H5-H4_ = 9.0 Hz, H^5^), 4.36 (dd, 1H, *J*_H6a-H5_ = 5.0 Hz, *J*_H6a-H6b_ = 12.0 Hz, H^6a^), 4.41 (dd, 1H, *J*_OH6′-H6′a_ ≈ *J*_OH6′-H6′b_ = 6.0 Hz, OH^6′^), 4.47 (dd, 1H, *J*_H6b-H5_ = 1.5 Hz, *J*_H6b-H6a_ = 12.0 Hz, H^6b^), 4.68 (d, 1H, *J*_OH3′-H3′_ = 8.0 Hz, OH^3′^), 4.85 (dd, 1H, *J*_OH1′-H1′a_ ≈ *J*_OH1′-H1′b_ = 6.5 Hz, OH^1′^), 4.96 (d, 1H, *J*_OH3-H3_ = 5.0 Hz, OH^3^), 5.17 (d, 1H, *J*_OH4-H4_ = 5.0 Hz, OH^4^), 5.18 (d,1H, *J*_OH2-H2_ = 6.0 Hz, OH^2^), 5.21 (d, 1H, *J*_OH4′-H4′_ = 5.5 Hz, OH^4′^), 5.24 (d, 1H, *J*_H1-H2_ = 3.5 Hz, H^1^), 7.42–7.46 (m, 1H, ArH), 7.50–7.54 (m, 2H, ArH), 7.74–7.76 (m, 2H, ArH), 7.82–7.85 (m, 2H, ArH), 8.05–8.08 (m, 2H, ArH) ppm. ^13^C NMR (DMSO-*d*_6_): δ = 62.5, 63.0, 64.8, 70.5, 70.6, 72.0, 73.1, 74.9, 77.3, 83.1, 92.2, 104.5, 127.4, 127.5, 128.9, 129.0, 129.6, 130.4, 139.4, 145.1, 166.1 ppm. HRMS (ESI) m/z for C_25_H_30_NaO_12_ [M + Na]^+^: calcd 545.1630; found 545.1636.

β-d-Fructofuranosyl 6-*O*-(2-phenylethanoyl)-α-d-glucopyranoside, sucrose phenyl acetate (**3k**, URB1480) [46].

White solid. Yield: 50%. MS (ESI): 459 [M–H]^−^, 478 [M + NH_4_]^+^, 483 [M + Na]^+^. ^1^H NMR (DMSO-*d*_6_): δ = 3.07 (ddd, 1H, *J*_H4-OH4_ = 6.0 Hz, *J*_H4-H3_ ≈ *J*_H4-H5_ = 9.5 Hz, H^4^), 3.21 (ddd, 1H, *J*_H2-H1_ = 3.5 Hz, *J*_H2-OH2_ = 6.0 Hz, *J*_H2-H3_ = 9.5 Hz, H^2^), 3.39–3.43 (m, 2H, H^1′a^, H^1′b^), 3.50 (ddd, 1H, *J*_H3-OH3_ = 5.0 Hz, *J*_H3-H2_ ≈ *J*_H3-H4_ = 9.5 Hz, H^3^), 3.58–3.64 (m, 3H, H^5′^, H^6′a^, H^6′b^), 3.67 (d, *J* = 16.0 Hz, 1H, *H*CHAr), 3.72 (d, *J* = 16.0 Hz, 1H, HC*H*Ar), 3.76–3.82 (m, 1H, H^4′^), 3.90 (dd, 1H, *J*_H3′-OH3′_ ≈ *J*_H3′-H4′_ = 8.0 Hz, H^3′^), 3.95 (m, 1H, H^5^), 4.05 (dd, 1H, *J*_H6a-H5_ = 6.0 Hz, *J*_H6a-H6b_ = 11.5 Hz, H^6a^), 4.28 (dd, 1H, *J*_H6b-H5_ = 1.5 Hz, *J*_H6b-H6a_ = 11.5 Hz, H^6b^), 4.43 (dd, 1H, *J*_OH6′-H6′a_ ≈ *J*_OH6′-H6′b_ = 5.5 Hz, OH^6′^), 4.60 (d, 1H, *J*_OH3′-H3′_ = 8.0 Hz, OH^3′^), 4.83 (dd, 1H, *J*_OH1′-H1′a_ ≈ *J*_OH1′-H1′b_ = 6.5 Hz, OH^1′^), 4.90 (d, 1H, *J*_OH3-H3_ = 5.0 Hz, OH^3^), 5.03 (d, 1H, *J*_OH4-H4_ = 6.0 Hz, OH^4^), 5.13 (d, 1H, *J*_OH2-H2_ = 6.0 Hz, OH^2^), 5.19 (d, 1H, *J*_H1-H2_ = 3.5 Hz, H^1^), 5.20 (d, 1H, *J*_OH4′-H4′_ = 6.0 Hz, OH^4′^), 7.24–7.35 (m, 5H, ArH) ppm. ^13^C NMR (DMSO-*d*_6_): δ = 21.2, 62.7, 63.1, 64.6, 70.6, 72.0, 73.1, 75.0, 77.4, 83.2, 92.0, 104.4, 127.2, 128.7, 129.9, 134.8, 171.7 ppm.

β-d-Fructofuranosyl 6-*O*-[2-(4-phenyl)phenylethanoyl]-α-d-glucopyranoside, sucrose *p*-biphenyl acetate (**3l**, URB1482).

White solid. Yield: 58%. MS (ESI): 527 [M–H]^–^, 536 [M + NH_4_]^+^, 541 [M + Na]^+^. ^1^H NMR (DMSO-*d*_6_): δ = 3.03 (ddd, 1H, *J*_H4-OH4_ = 6.0 Hz, *J*_H4_-_H3_ ≈ *J*_H4-H5_ = 9.5 Hz, H^4^), 3.13 (ddd, 1H, *J*_H2-H1_ = 3.5 Hz, *J*_H2-OH2_ = 6.0 Hz, *J*_H2-H3_ = 9.5 Hz, H^2^), 3.37–3.41 (m, 2H, H^1′a^, H^1′b^), 3.48 (ddd, 1H, *J* _H3-OH3_ = 5.0 Hz, *J*_H3-H2_ ≈ *J*_H3-H4_ = 9.5 Hz, H^3^), 3.56–3.63 (m, 3H, H^5′^, H^6′a^, H^6′b^), 3.76–3.81 (m, 1H, H^4′^), 3.88–3.96 (m, 2H, H^3′^, H^5^), 4.14 (dd, 1H, *J*_H6a-H5_ = 5.0 Hz, *J*_H6a-H6b_ = 11.5 Hz, H^6a^), 4.33 (dd, 1H, *J*_H6b-H5_ = 1.0 Hz, *J*_H6b-H6a_ = 11.5 Hz, H^6b^), 4.42 (dd, 1H, *J*_OH6′-H6′a_ ≈ *J*_OH6′-H6′b_ = 5.5 Hz, OH^6′^), 4.62 (d, 1H, *J*_OH3′-H3′_ = 8.0 Hz, OH^3′^), 4.82 (dd, 1H, *J*_OH1′-H1′a_ ≈ *J*_OH1′-H1′b_ = 6.0 Hz, OH1′), 4.89 (d, 1H, *J*_OH3-H3_ = 5.0 Hz, OH^3^), 5.02 (d, 1H, *J*_OH4-H4_ = 6.0 Hz, OH^4^), 5.13 (d, 1H, *J*_OH2-H2_ = 6.0 Hz, OH^2^), 5.17 (d, 1H, *J*_H1-H2_ = 3.5 Hz, H^1^) 5.20 (d, 1H, *J*_OH4′-H4′_ = 5.5 Hz, OH^4′^), 7.24–7.28 (m, 2H, ArH), 7.29–7.37 (m, 7H, ArH) ppm. ^13^C NMR (DMSO-*d*_6_): δ = 56.1, 62.5, 63.0, 64.6, 70.3, 70.4, 71.9, 73.1, 75.0, 77.4, 83.2, 92.0, 104.5, 127.4, 127.5, 128.9, 130.0, 139.48, 139.52, 172.4 ppm. HRMS (ESI) m/z for C_26_H_32_NaO_12_ [M + Na]^+^: calcd 559.1786; found 559.1790.

β-d-Fructofuranosyl 6-*O*-[2-(4,4′-biphenyl)phenylethanoyl]-α-d-glucopyranoside, sucrose *p*-triphenylacetate (**3m**, URB1543).

White solid. Yield: 31%. MS (ESI): 611 [M–H]^−^, 630 [M + NH4]^+^, 635 [M + Na]^+^.^1^H NMR (DMSO-*d*_6_) δ: 3.08 (ddd, 1H, *J*_H4-OH4_ = 5.0 Hz, *J*_H4_-_H3_ ≈ *J*_H4-H5_ = 9.5 Hz, H4), 3.23 (ddd, 1H, *J*_H2-H1_ = 4.0 Hz, *J*_H2-OH2_ = 6.0 Hz, *J*_H2-H3_ = 9.5 Hz, H2), 3.38–3.43 (m, 2H, H1′a, H1′b), 3.46–3.54 (m, 1H, H3), 3.57–3.66 (m, 3H, H5′, H6′a, H6′b), 3.73 (d, *J* = 16.0 Hz, 1H, *H*CHAr), 3.77 (d, *J* = 16.0 Hz, 1H, HC*H*Ar), 3.79–3.82 (m, 1H, H4′), 3.90 (dd, 1H, *J*_H3′-OH3′_ ≈ *J*_H3′-H4′_ = 8.0 Hz, H3′), 3.97 (m, 1H, H5), 4.08 (dd, 1H, *J*_H6a-H5_ = 6.0 Hz, *J*_H6a-H6b_ = 11.5 Hz, H6a), 4.31 (dd, 1H, *J*_H6b-H5_ = 1.5 Hz, *J*_H6b-H6a_ = 11.5 Hz, H6b), 4.46 (dd, 1H, *J*_OH6′-H6′a_ ≈ *J*_OH6′-H6′b_ = 5.5 Hz, OH6′), 4.62 (d, 1H, *J*_OH3′-H3′_ = 8.0 Hz, OH3′), 4.83 (dd, 1H, *J*_OH1′-H1′a_ ≈ *J*_OH1′-H1′b_ = 6.0 Hz, OH1′), 4.84 (brs, 1H, OH3), 5.06 (d, 1H, *J*_OH4-H4_ = 5.0 Hz, OH4), 5.15 (d, 1H, *J*_OH2-H2_ = 6.0 Hz, OH2), 5.21 (m, 2H, H1, OH4′), 7.35–7.42 (m, 3H, ArH), 7.45–7.51 (m, 2H, ArH), 7.66–7.78 (m, 8H, ArH) ppm. ^13^C NMR (DMSO-*d*_6_) δ: 62.7, 63.1, 64.7, 70.5, 70.6, 72.0, 73.1, 75.0, 77.3, 83.2, 91.9, 104.4, 126.9, 127.0, 127.5, 127.55, 127.65, 128.0, 129.4, 130.6, 134.2, 138.5, 139.3, 139.6, 140.1, 171.7 ppm. HRMS (ESI) m/z for C_32_H_36_NaO_12_ [M + Na]^+^: calcd 635.2099; found 635.2104.

### 3.3. Bacterial Strains and Culture Conditions

Eight reference human pathogens were used in this study: *E. faecalis* ATCC 29212, *E. coli* O157:H7 ATCC 35150, *S. aureus* ATCC 43387, *S. aureus* ATCC 43300 (MRSA), *L. monocytogenes* ATCC 7644, *K. pneumoniae* ATCC 13883, *P. aeruginosa* ATCC 9027, and *S. enteritidis* ATCC 13076. All the strains were maintained in TSA (tryptone soy agar) (VWR, Milan, Italy) at 37 °C, while the stock cultures were kept at −80 °C in nutrient broth with glycerol 15%.

### 3.4. Fungi and Culture Conditions

Three pathogenic filamentous fungi belonging to the strain collection of Pharmacology and Hygiene Section (Department of Biomolecular Sciences, University of Urbino Carlo Bo) *A. niger* ATCC 9642, *A. fumigatus* IDRAH01 and *Fusarium* spp., as well as the reference strain *C. albicans* ATCC 10231, were included. All the filamentous fungi were maintained on potato dextrose agar (PDA) (VWR) at 30 °C for 7 days, while *C. albicans* was grown at 37 °C for 24 h.

### 3.5. Minimum Inhibitory Concentration (MIC)

MIC of each compound was determined by standard micro-dilution method according to the National Committee for NCCLS (Clinical Laboratory Standards) document M100-S12 method (CLSI, 2016). First, stock solution of each compound (~12 mg) was prepared in 50:50 (*v*/*v*) of distilled sterile water and biological grade DMSO (final volume 1 mL). Several colonies of each bacterial strain were inoculated in 10 mL of sterile Mueller–Hinton broth II (MHB II) (VWR) and incubated at 37 °C for 24 h. At the end of the incubation period, each suspension was diluted in MHB II to obtain ca. 10^5^ cfu/mL and 100 µL was added in wells of the 96-well plate together with the appropriate volumes of the test solutions (final concentration range: 4–1024 µg/mL). Two rows were used for positive (bacteria alone) and negative controls (MHB II alone), respectively. Preliminary assays with DMSO were carried out to exclude its possible bacteriostatic and/or bactericidal activity; in any case, the quantity of DMSO added in each well never exceeded 5% (*v*/*v*) of the final total volume. MIC was defined as the lowest concentration of compound able to inhibit bacterial growth after 24 h of incubation at 37 °C. All the experiments were performed in duplicate.

Regarding the filamentous fungi, spores were harvested from PDA plates by adding 2 mL of sterile 0.85% saline solution supplemented with 0.05% Tween 80; the surface was scraped with a sterile cotton swab and the suspension, transferred in a sterile tube, was left at room temperature for 5 min to allow the sedimentation of hyphal fragments. The upper homogeneous suspension was transferred into a new sterile tube, vortexed for 15 s and adjusted to an optical density (OD 530 nm) between 0.09 to 0.4 (about 10^6^ spores/mL). For *C. albicans* the suspension was adjusted with the spectrophotometer to a turbidity of 0.12 (about 10^6^ cfu/mL). Successively, 100 μL of each fungal suspension was diluted 1:50 in standard RPMI 1640 medium (Sigma-Aldrich, Milan, Italy) and inoculated into 96-well plates together with the appropriate volumes of each test solution as described above. Two rows were left for positive control growth and negative controls (medium only), respectively. Plates were incubated at 30 °C for 72 h; in the case of *C. albicans* plates were incubated at 37 °C for 24–48 h. MIC is defined as the lowest drug concentration that inhibits visible growth compared to the untreated control. In addition, the turbidity of the 96-wells plate was assessed by spectrophotometer (530 nm) (Multiskan EX, Thermo Scientific, Waltham, MA, USA).

### 3.6. DPPH Assays

The antioxidant capacity of URB1537 was evaluated in a cell-free system by the DPPH radical scavenging assay, as previously described (Verboni 2022). Concentrations up to 512 µg/mL were tested, and the EC_50_ value (i.e., the concentration necessary to achieve a 50% antioxidant effect) was calculated. Quercetin (up to 4.5 µg/mL) was used as a reference antioxidant molecule to check the procedure’s correctness.

### 3.7. Cytotoxicity Assay

The cytotoxicity of URB1537 was investigated on HaCaT cells (immortalized human keratinocytes) from CLS-Cell Lines Service GmbH (Eppelheim, Germany) in vitro. Cells (5 × 10^3^/well) were seeded in 96-well plates and incubated for 24 h with S-C16 (16–512 µg/mL). After incubation, cell viability was analyzed by the MTT assay. Color development was monitored at 570 nm in a multi-well plate reader (Thermo Scientific, Waltham, MA, USA), and data were expressed as cell viability (%) vs. untreated control cells. The IC_50_ value (i.e., the concentration required to reduce cell viability by 50%) was then calculated.

### 3.8. Griess Assay

The anti-inflammatory properties of URB1537 were evaluated in RAW 264.7 cells (murine macrophages) stimulated by lipopolysaccharide (LPS) (Sigma-Aldrich, Milan, Italy). Cells (3 × 10^4^/well) were seeded in 96-well plates and treated for 24 h with LPS (1 µg/mL) in the presence of URB1537 (16–128 µg/mL). After that, NO release was determined in the culture medium by the Griess reagent (Sigma-Aldrich, Milan, Italy), as recently reported (Verboni 2022). The anti-inflammatory effects of URB1537 (128 µg/mL) in comparison to sucrose and palmitoleic acid were also assessed. Dexamethasone (2 µg/mL) was used as a reference anti-inflammatory drug. RAW 264.7 cell viability after LPS, URB1537, and dexamethasone administration were assessed by the MTT assay as described above.

### 3.9. Statistical Analysis

Comparisons between multiple means were performed via ANOVA followed by Tukey’s post hoc test. Significance was set at *p* < 0.05. GraphPad Prism 6.0 (GraphPad Software, Inc., San Diego, CA, USA) was used for statistical analysis.

## 4. Conclusions

An effective variation of a Mitsunobu-type methodology reported in the literature has allowed obtaining a small library of 6-*O*-sucrose-based surfactants. The ameliorated procedure proved to be versatile and to have a large scope, starting from non-protected and commercially available substrates. The compounds were then screened for antimicrobic and antimycotic activities. Interestingly, some of the herein-reported sucrose monoesters showed interesting antifungal activities. In particular, sucrose palmitoleate (**3d**, URB1537) shows promising antifungal activity against *C. albicans*, *Fusarium* spp. and *A. fumigatus* with MIC values of 16, 32, and 64 μg/mL, respectively. For this reason, URB1537 was selected for further biological characterizations. The new surfactant is shown to be safe for human keratinocytes and murine macrophages up to 128 µg/mL. Although URB1537 did not show any antioxidant properties within the range of tested concentrations (up to 512 µg/mL), 6-*O*-sucrose palmitoleate presents an appreciable anti-inflammatory activity in LPS-activated macrophages, suggesting that the compound might help to control the inflammatory response. Thus, URB1537 could have therapeutic potential and could also be used in designing and developing innovative drugs such as those based on liposomes.

## Figures and Tables

**Table 1:** Calculated physicochemical properties of the sucrose-based ester surfactants.

Entry	Sucrose Ester	MW	HLB ^a^	LogP ^b^
1	Caprylate C8	468.5	12.7	−1.4
2	Undecylenate C11:1	508.6	11.7	−0.2
3	Undecylinate C11:1	506.6	11.8	−0.9
4	Palmitoleate C16:1	578.7	10.3	2.0
5	Oleate C18:1	606.7	9.8	2.9
6	Linoleate C18:2	604.7	9.9	2.6
7	Linolenate C18:3	602.7	9.9	2.4
8	Nervonate C24:1	690.9	8.6	5.9
9	Benzoate	446.4	13.4	−2.7
10	*p*-Phenyl benzoate	522.5	11.4	−0.3
11	Phenyl acetate	460.4	12.9	−2.1
12	*p*-Biphenyl acetate	536.5	11.1	−0.4
13	*p*-Triphenyl acetate	612.6	9.7	0.6

^a^ Calculated hydrophilic–lipophilic balance by Griffin’s method for non-ionic surfactants [33] [HLB = 20 × (MW hydrophilic portion/MW)]. ^b^ Calculated octanol–water portion coefficient clogP (by OSIRIS Property Explorer) [34].

**Table 2:** Investigation of the conditions for Mitsunobu-type reaction between sucrose and undecylenic acid.

Entry ^a^	Eq. 2b	Eq. DIAD:PPh_3_	T (°C)	t (h)	Dry Solvent	Yield (%) 3b ^b^	Yield (%) 4 ^b^
1	2.5	2.7:2.7	20	24	DMF	34	38
2	1	2.5:2.5	20	24	DMF	19	trace
3	1.5	2.5:2.5	20	24	DMF	39	15
4	1.5	1.5:1.5	20	24	DMF	26	trace
5	1	1:1	20	24	DMF	trace	-
6	1.5	3:3	20	24	DMF	39	16
7	1.5	2.5:2.5	60	24	DMF	trace	41
8	1.5	2.5:2.5	20	6	DMF	12	2
9	1.5	2.5:2.5	20	12	DMF	18	9
10	1.5	2.5:2.5	20	24	THF	trace	-
11	1.5	2.5:2.5	20	24	dioxane	trace	-
12 ^c^	1.5	2.5 ^c^:2.5	20	24	DMF	35	19
13 ^d^	1.5	2.5 ^d^:2.5	20	24	DMF	19	trace

^a^ Reaction conditions: reactions were carried out under nitrogen atmosphere with **1** (1.0 mmol, 1 eq.), and **2b** (x eq.), DIAD (y eq.), PPh_3_ (y eq.) and solvent (10 mL) at T (°C) for certain time, t (h). ^b^ Yields are referred to isolated products. ^c^ Reaction was performed using DEAD. ^d^ Reaction was performed using DCAD. Eq. = equivalent.

**Table 3:** Antibacterial activity of sucrose-based compounds (MIC values are reported in µg/mL).

Specie Target	3a	3b	3c	3d	3e	3f	3g	3h	3i	3j	3k	3l	3m
*E. faecalis* ATCC 29212	>1024	>1024	1024	1024	1024	256	512	1024	1024	1024	1024	>1024	>1024
*E. coli* O157:H7 ATCC 35150	>1024	>1024	>1024	>1024	>1024	>1024	>1024	>1024	>1024	>1024	>1024	>1024	>1024
*S. aureus* ATCC 29213	>1024	>1024	>1024	1024	1024	1024	>1024	>1024	>1024	>1024	>1024	>1024	>1024
*S. aureus* ATCC 43300	>1024	>1024	>1024	1024	1024	1024	>1024	>1024	>1024	>1024	>1024	>1024	>1024
*K. pneumoniae* ATCC 13883	>1024	>1024	>1024	>1024	>1024	>1024	>1024	>1024	>1024	>1024	>1024	>1024	>1024
*L. monocytogenes* ATCC 7644	>1024	>1024	1024	1024	1024	1024	1024	>1024	>1024	1024	>1024	>1024	>1024
*S. enteritidis* ATCC 13076	>1024	>1024	>1024	>1024	>1024	>1024	>1024	>1024	>1024	>1024	>1024	>1024	>1024
*P. aeruginosa* ATCC 9027	>1024	>1024	>1024	>1024	>1024	>1024	>1024	>1024	>1024	>1024	>1024	>1024	>1024

**Table 4:** Antifungal activity of sucrose-based compounds (MIC values are reported in µg/mL).

Specie Target	3a	3b	3c	3d	3e	3f	3g	3h	3i	3j	3k	3l	3m
*A. fumigatus* IDRAH01	1024	1024	>1024	64	16	32	1024	1024	>1024	>1024	1024	>1024	>1024
*A. niger* ATCC 9642	1024	512	>1024	512	1024	1024	>1024	>1024	>1024	>1024	>1024	>1024	>1024
*Fusarium* spp.	>1024	512	1024	32	128	128	128	1024	512	1024	256	1024	>1024
*C. albicans* ATCC 10231	1024	1024	1024	16	1024	1024	512	>1024	256	1024	1024	1024	>1024

## Data Availability

Data are contained within the article.
